# Above and beyond the Lab Scale—Creating a kW-Sized AEM Electrolyzer Validated by In-Situ Distribution of Relaxation Times

**DOI:** 10.1021/acs.energyfuels.5c03702

**Published:** 2025-09-30

**Authors:** Suhas Nuggehalli Sampathkumar, Thomas Benjamin Ferriday, Zoé Mury, Philippe Aubin, Khaled Lawand, Jan Van Herle

**Affiliations:** † Group of Energy Materials, 27218 École Polytechnique Fédérale de Lausanne (EPFL), Rue de l’Industrie 17, Sion 1951, Valais Switzerland; ‡ Centre for Materials Science and Nanotechnology, 6305 University of Oslo, Gaustadalléen 21, Oslo 0349, Norway

## Abstract

Most reported anion exchange membrane water electrolyzers (AEMWEs) are currently limited to the usual 1–10 cm^2^ electrodes in single-cell AEMWEs; however, accelerating its technology readiness level necessitates an explosive increment in unit sizes. We report the design, characterization, and validation of a 1 kW, 500 cm^2^ (5 × 100 cm^2^) non-PGM AEMWE stack. Complete with corrosion protection, internal heating, and a control system, the patented stack design operated stably at 1.0 A cm^–2^ with an energy efficiency of 53.2 kWh kg_H_2__^–1^. Moreover, to confirm cell-to-cell uniformity, a comprehensive statistical analysis was carried out to reveal five uniformly performing cells. Impedance analysis complemented by distribution of relaxation times (DRT) analysis revealed kinetic insights similar to those traditionally obtained for lab-scale electrodes, proving both non-PGM electrode scalability and efficacy, and the utility of DRT analysis on large-scale AEMWE stacks.

## Graphical abstract

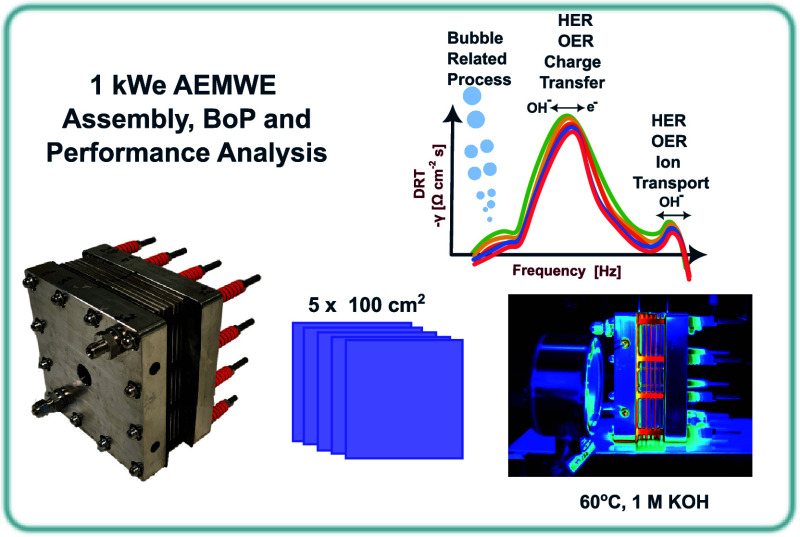

The green energy shift is heavily invested in various hydrogen technologies, where anion exchange membrane water electrolyzer (AEMWE) technology is a key player.^1–3^ AEMWEs offer great benefits through material scalability and transferable know-how from mature industries, which has accelerated their transition from lab-scale to industry. However, the current energy consumption for state-of-the-art industrial AEMWEs lies around 53 kWh kg_H_2__^–1^, which must be further lowered to 48 kWh kg_H_2__^–1^ to meet the Strategic Research and Innovation Agenda (SRIA) 2030 target.^2^

Development of efficient catalyst materials is necessary to meet this target, although most are insufficiently tested, as their stack performance is ultimately the only important parameter. This emphasizes the urgency of stack optimization, where sparse literature^4–9^ shows that few units reach 1 kW.^5–8^

While these studies are promising in demonstrating the scalability of AEMWE technology, they do not provide a detailed analysis of internal loss mechanisms. In particular, they lack comprehensive electrochemical impedance spectroscopy (EIS) investigations as a function of current density under relevant operating conditions, an essential diagnostic tool for understanding and optimizing stack performance.

Factors such as cell-to-cell variation, the influence of manifold design on liquid distribution and two-phase flow evacuation, and the impact of thermal gradients on localized performance are effectively mapped using EIS. These insights are crucial for design improvements and will be essential to meet the SRIA 2030 target.

## Results and Discussion

A detailed material analysis was featured in previous work,^10^ and here a 5-cell, 500 cm^2^ non-PGM AEMWE stack was created with the same materials and evaluated under galvanostatic operation to determine its performance characteristics and assess its potential for further scale-up (10 kW). This investigation focused on stack-level response under increasing current loads, aiming to validate efficiency, power input, and cell uniformity. Particular attention was given to identifying stable operating regions and understanding how closely the performance of this nonprecious metal configuration aligns with industrial benchmarks.

Figure 1a shows the linear sweep voltammogram (LSV) for the five cells, where all cells maintain approximately 1.0 A cm^–2^ at 2.0 V. A thorough statistical analysis provided in the Supporting Information shows a homogeneous performance for all cells, although cell 3 yielded the most even performance, based on several statistical descriptors.

**Figure 1:**
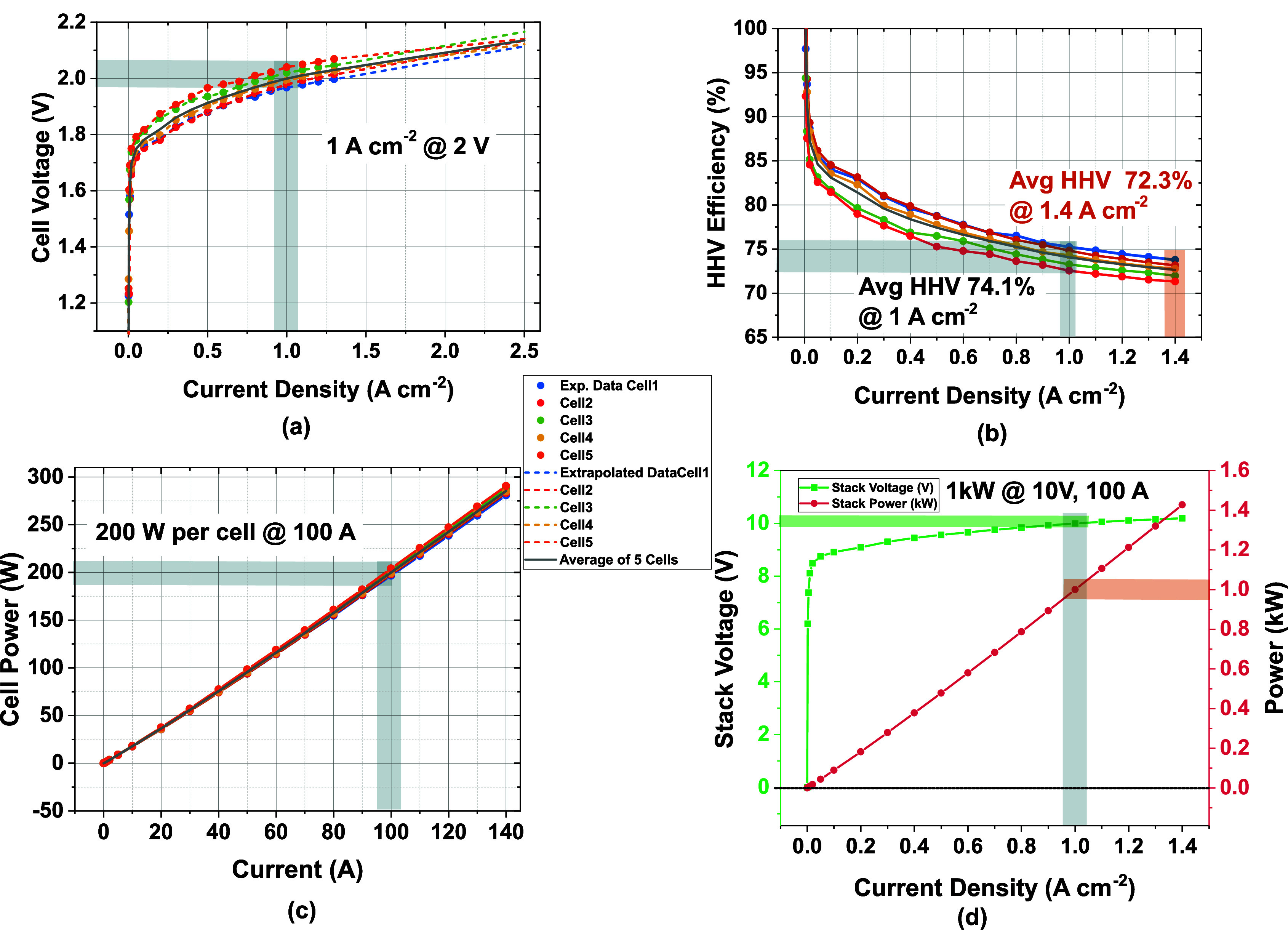
Performance characterization of the 5-cell non-PGM AEMWE stack: (a) current density–voltage curves without ohmic compensation; (b) higher heating value (HHV) efficiency, highlighting peak and nominal values; (c) power output of individual cells; and (d) resulting total stack power.

This consistency points to even current distribution, effective MEA integration, and minimal variation in cell hardware/thermal conditions. The nominal operating point of 1.0 A cm^–2^ at approximately 2.0 V per cell is highlighted as a stable and representative condition. Beyond the experimental range, extrapolated data points (shown as dashed extensions) provide a theoretical view of the performance at higher current densities.

More importantly, Figure 1b shows that the HHV-based efficiency at 1.0 A cm^–2^ (2.0 V) is 74.1%, which translates to an energy consumption of approximately 53.2 kWh kg_H_2__^–1^, approximately meeting the SRIA 2024 target of 53 kWh kg_H_2__^–1^.^2^ While these values reflect competitive performance for a non-PGM system operating under alkaline conditions, they remain above the 2030 SRIA target of 48 kWh kg_H_2__^–1^, underscoring the need for further reductions in overpotentials and enhanced system integration to meet future efficiency benchmarks.^2^ At a current density of 1.0 A cm^–2^, the AEMWE stack currently exceeds the 2030 target by 10.83%. Bridging this efficiency gap will require continued advancements in materials development and stack architecture, with a particular focus on minimizing voltage losses under high-current operation.

To understand how the stack scales in terms of energy delivery, Figure 1c plots the cell power as a function of stack current. The relationship is linear across the entire tested range, suggesting that the system operates predominantly in the ohmic regime, with no significant mass transport or kinetic limitations up to 140 A/cell. Each cell adds approximately >260 W at this current, yielding a combined stack input of 1.43 kW_e_. This linearity in power output not only reflects electrical predictability but also reinforces the quality of system integration and thermal management.

Finally, the overall stack performance is summarized in Figure 1d, which presents total stack voltage and stack power against current density. As current increases, the stack voltage rises nonlinearly, consistent with the accumulation of individual cell overpotentials. Meanwhile, the stack power increases linearly, peaking at around 1.4 kW. The shaded region identifies a nominal operating window where efficiency, durability, and power output are all perfectly balanced. Operation at 1.0 A cm^–2^ results in a stack output of approximately 1.0 kW at 10 V, thereby confirming alignment with the performance requirements of a 1.0 kW_e_-class electrolyzer.

The EIS spectra in Figure 2a demonstrate uniform polarization impedances across the five-cell AEMWE stack, with Nyquist plots revealing a consistent semicircular profile that persists across increasing current densities. At 1.0 A cm^–2^, a significant shift in the ohmic resistance is observed, attributed to Joule heating. The absence of a pronounced high-frequency inductive loop further confirms the integrity of the cell configuration and the efficacy of the cabling layout, particularly in terms of minimizing parasitic inductance and ensuring precise placement of current and voltage taps. The quality of the impedance data is substantiated by Kramers–Krönig tests, with residuals within ±1.5%.

**Figure 2:**
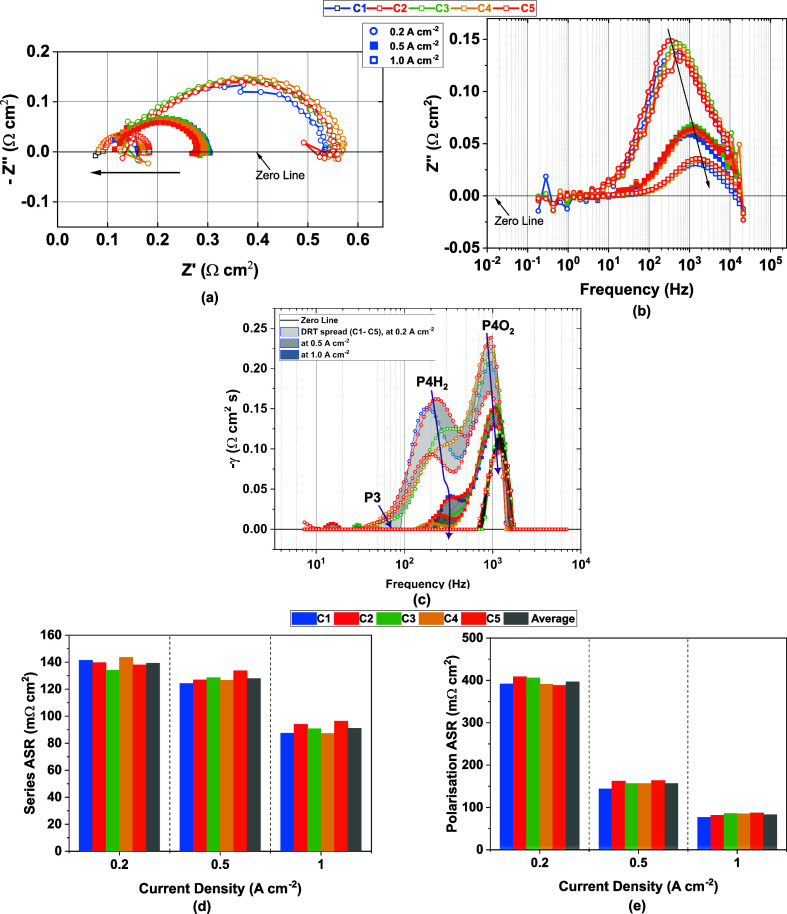
Electrochemical characterization of the 5-cell non-PGM AEMWE stack: (a) Nyquist plots from EIS measurements at 0.2, 0.5, and 1.0 A cm^–2^; (b) Bode plots (imaginary impedance vs frequency) showing a reduction in charge transfer resistance; (c) DRT spectra across cells C1–C5, revealing more uniform behavior at higher current densities; and (d) series and polarization resistance of the stack.

Figure 2b presents the frequency distribution of the imaginary component, elucidating the frequency response of the charge transfer processes. An increase in current density markedly lowers the charge transfer resistance and shifts it to higher frequencies, implying faster kinetics with lower time constants. Additionally, the emergence of a low-frequency inductive loop, especially at 0.2 A cm^–2^, suggests dynamic behavior associated with drift phenomena or interfacial effects under transient input power conditions. Further studies are deemed necessary to understand this low-frequency behavior in AEMWE systems.

This inductive feature has recently gained attention, where several authors^11–13^ have investigated the phenomena. Their findings reveal such behavior may enhance DC efficiency, as the slope of the *i–V* curve (representing DC resistance) falls below the high-frequency resistance, thereby improving performance. This insight opens a promising avenue for AEMWE systems, suggesting that it is not merely an artifact but potentially a beneficial operational mechanism.

The DRT analysis of the stack in Figure 2c reveals several distinct features that elucidate the interplay between the kinetic and transport processes. The processes identified in our previous work have been summarized in the Supporting Information (Table S4). At low current densities, a small shoulder peak appears between 40 and 80 Hz, accompanied by two prominent peaks near 208 and 900 Hz. As the current density increases, the amplitude of these major peaks declines and the shoulder peak disappears. This behavior is characteristic of charge transfer processes, which typically decrease as overpotentials increase. The shoulder aligns with peak P3, previously assigned in prior work^10^ to gas and water diffusion within the HER and OER electrodes.

The first prominent peak, at approximately 208 Hz, is primarily attributed to charge transfer of the hydrogen evolution reaction (HER), and its sharp decline supports this interpretation. However, given the coupled nature of the electrochemical reactions and the shared ionic environment in the stack, it is likely that this peak also contains partial contributions from the OER-related processes. The OER-related processes overlap with the HER charge transfer,^10,14^ even without the presence of mass-transfer-related resistance (P3). Conversely, the second peak around 900 Hz is dominated by OER charge transfer, but may similarly include overlapping features associated with HER. This bidirectional overlap is consistent with observations from prior work on the same materials from the lab-scale single-cell level,^10^ which associated the 208 Hz peak with P4H_2_ and the 900 Hz peak with P4O_2_, both exhibiting some spectral blending.

Conversely to prior work,^10^ decoupling P4O_2_ into individual subprocesses such as P4­(i)­O_2_ and P4­(ii)­O_2_ is not feasible with the current dataset, due to the extent of overlap and resolution limitations.

The absence of the typical high-frequency P5 peak attributed to OH^–^ ion transport between HER and the OER regions suggests that this process is either suppressed or obscured in the stack environment. This is likely a consequence of geometric surface area effects, interfacial heterogeneity, and the distributed nature of ion conduction across the membrane–electrode interfaces in large-area cells. As there is currently no literature available to validate this hypothesis, single repeating unit experiments should be conducted, comparing the DRT obtained from smaller-area single-cell tests.

As the current density increases, the dispersion in DRT response between individual cells decreases, indicating that (i) cell-to-cell disparity is related to variable H_2_ kinetics and (ii) all cells have largely similar O_2_ kinetics. The P4 peak, observed around 208.78 Hz at 0.2 A cm^–2^, shifts to 305.42 Hz at 0.5 A cm^–2^ and vanishes by 1.0 A cm^–2^, highlighting its strong dependence on the current. In contrast, the P5 peak shifts from 886.07 to 1296.19 Hz while steadily decreasing in magnitude. Minor features appearing in the 5–20 Hz range for cells C3 and C5 at 0.5 A cm^–2^ are likely artifacts introduced by the smoothing algorithm, as they are absent in the corresponding Bode spectra. Figures 2d and 2e quantify the evolution of series and polarization ASR. The series resistance decreases by 34.6%, from an average of 139 mΩ cm^–2^ at 0.2 A cm^–2^ to 91 mΩ cm^–2^ at 1.0 A cm^–2^. In contrast, the polarization resistance reduces 79.2%, from 396.85 mΩ cm^–2^ to 82.87 mΩ cm^–2^.

Cell 3 was selected for further in-depth analysis because it captured neither the very best nor worst performance, thus serving as an average, middle-ground representation of the stack. This was confirmed by the statistical analysis in the Supporting Information (section 2.2).

The most substantial reduction in polarization resistance for the center cell, C3, occurs at low current densities, particularly between 0.05 and 0.1 A cm^–2^, as shown in Figure 3a. This initial drop signifies the rapid activation of electrochemical processes. As the current density increases from 0.1 to 1.0 A cm^–2^ (Figure 3b), the decline in polarization resistance becomes more gradual, indicating a transition from activation-dominated to ohmic and transport-influenced regimes.

**Figure 3:**
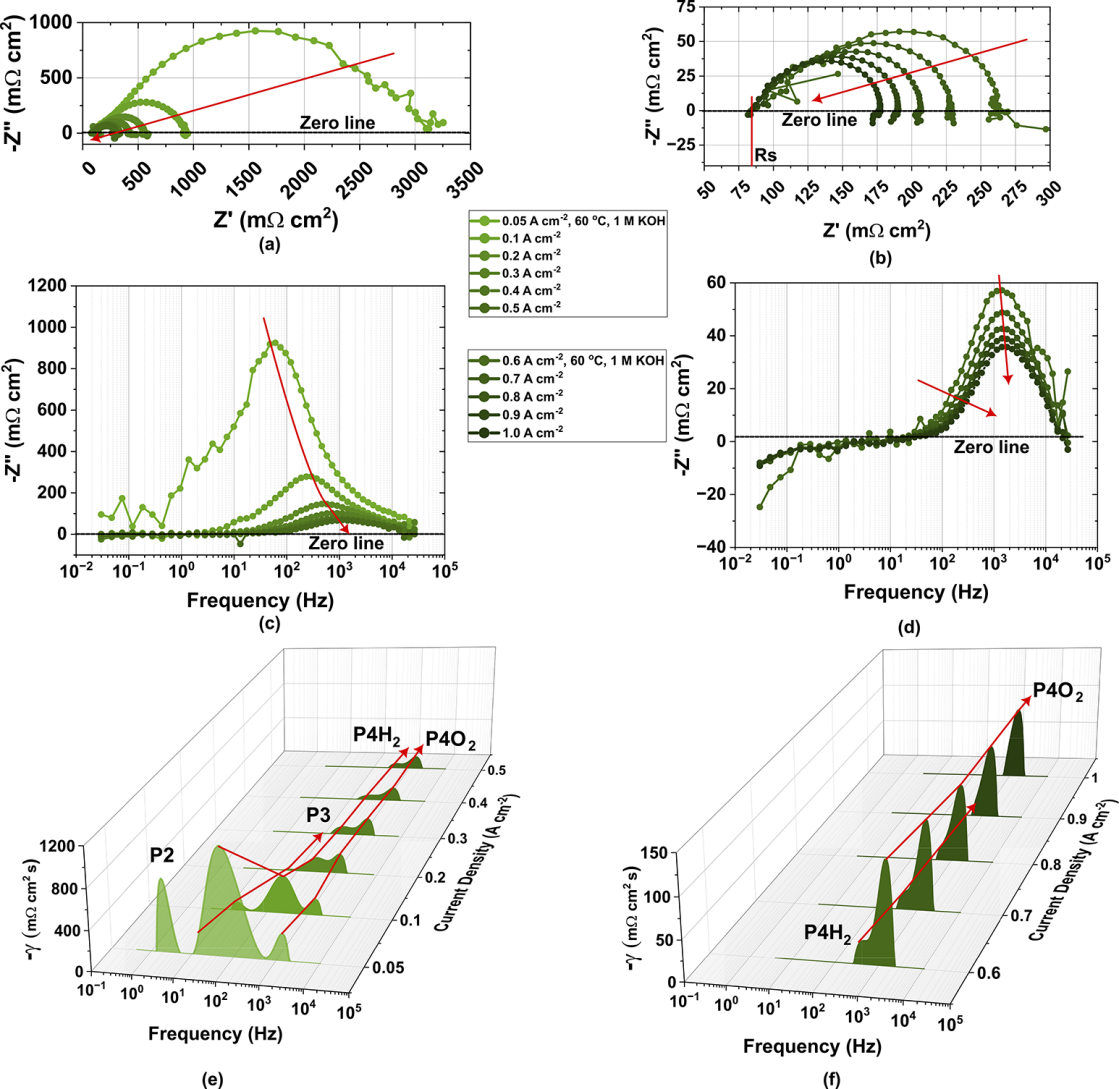
Electrochemical analysis of the center cell, C3: (a) and (b) Nyquist plots at current densities from 0.05 to 0.5 A cm^–2^ and 0.6 to 1.0 A cm^–2^, respectively; (c) and (d) the corresponding Bode plots, showing an increased peak frequency with rising current densities; (e and f) DRT spectra, further decoupling the MEA-associated mechanisms.

Low-frequency inductive loops begin to emerge at higher currents, although interpretation becomes limited due to noise in the so-called positive capacitance region, which affects the quality of the EIS data. The imaginary component of the Bode plots in Figures 3c and 3d mirrors the trends previously observed in Figure 3b, with a consistent increasing shift in peak frequency as the current density increases. This reflects improved charge transfer kinetics and reduced interfacial resistances under elevated operating conditions.

DRT spectra in Figures 3e and 3f further support this analysis. Similar to the total stack analysis in Figure 2, peak P4H_2_ diminishes notably in magnitude, consistent with the expected HER behavior, while P4O_2_ diminishes and stabilizes in both amplitude and frequency. P4O_2_ emerges as the dominant feature at higher current densities, suggesting that the OER is rate-limiting, as traditionally expected. As previously mentioned, P4O_2_ still exhibits contributions from P4H_2_. Decoupling these effects requires additional experiments involving comparative studies with and without the presence of an OER electrocatalyst, hence requiring further work. These trends align with performance-limiting mechanisms identified in PEMWE systems, underscoring the relevance of P4O_2_ as a critical descriptor of MEA performance degradation.

## Conclusion

This paper detailed a electrochemical performance and loss characterization of a five-cell, 500 cm^2^ non-PGM AEMWE stack, incorporating a patent-pending two-phase flow field and a consistent membrane-electrode assembly (Ni Fiber-Raney Ni ∥ X37–50RT ∥ NiFeO_*x*_-SS316L fiber). Operated at 60 °C in 1.0 M KOH, the system achieved a cold-start within seven min and a hot-idle recovery in one min, demonstrating effective thermal management and startup response.

At 1.0 A cm^–2^ and approximately 2.0 V per cell, the stack reached a HHV efficiency of 74.1%, corresponding to 53.2 kWh kg_H_2__^–1^ and delivering around 1.43 kW_e_, which is 10.83% short of the 2030 benchmark. EIS indicated a fairly uniform cell behavior across the stack. Statistical analysis showed that Cell 4 had the closest alignment with the mean stack voltage, while Cell 2 showed the largest deviations. Cell 3 was selected for further impedance analysis due to its intermediate response, representing the best and worst performing cells. The combined use of MAD, RMSD, and SD provided a clear assessment of both intercell and intracell voltage variations.

Increasing current density from 0.2 A cm^–2^ to 1.0 A cm^–2^ led to a 34.6% reduction in series resistance and a 79.2% drop in polarization resistance. These reductions are attributed to Joule heating and enhanced charge transfer kinetics at elevated current densities. DRT analysis resolved three distinct features: a shoulder between 40 and 80 Hz (P3), attributed to gas and water transport; and two dominant peaks, P4H_2_ (∼208 Hz) and P4O_2_ (∼900 Hz), associated with charge transfer during the hydrogen and oxygen evolution reactions. With an increase in current, all peaks decreased in magnitude and shifted to higher frequencies. The disappearance of P4 at a higher current density suggests reduced charge-transfer resistance in the porous electrodes. The stack exhibited increasingly uniform DRT responses across all cells at higher current densities. No clear peak linked to hydroxide ion transport was resolved (P5), likely due to geometric averaging and resolution limits in large-area assemblies.

In conclusion, this study demonstrates a consistent, scalable performance for non-PGM AEMWE stacks, with well-characterized losses and cell-to-cell uniformity. Future work should focus on isolating P4-related interfacial processes, addressing P3 transport resistances, and enhancing diagnostic resolution to enable detection of high-frequency phenomena such as P5, HER-OER ion transport, and further investigations of low-frequency inductive features. These steps will support progress toward meeting the SRIA 2030 targets (Supporting Information, References).

## Supplementary Material



## Notes

Experimental methods featuring an experimental section, system operations, electrochemical and statistical analysis; additional results including the impressed current cathodic protection and statistical analysis of the individual cells within the stack (PDF)
