# Supplementary material for: Above and beyond the Lab Scale—Creating a kW-Sized AEM Electrolyzer Validated by In-Situ Distribution of Relaxation Times
Source: Energy Fuels. 2025 Sep 30;39(40):19544–9. doi: 10.1021/acs.energyfuels.5c03702 (PMC12519592; doi:10.1021/acs.energyfuels.5c03702)
Supplement: Supplementary file 1 [file ef5c03702_si_001.pdf]

Supporting Information

Above and beyond the lab-scale - Creating a kW-sized AEM electrolyser validated by in-situ distribution of relaxation times

Suhas Nuggehalli Sampathkumar[*,†], Thomas Benjamin Ferriday[‡], Zoé Mury[†], Philippe Aubin[†], Khaled Lawand[†] and Jan Van Herle[†]

[†] Group of Energy Materials, Swiss Federal Institute of Technology, Lausanne, Rue de l'Industrie 17, Sion, 1951 Valais, Switzerland

[‡] Centre for Materials Science and Nanotechnology, University of Oslo, Gaustadalléen 21, 0349 Oslo, Norway

[*] Corresponding author; E-mail: suhas.nuggehalli@epfl.ch

1. Experimental

1.1 AEM stack components

The interior components of the AEMWE stack were scaled up versions of the same MEA components employed in our previous work.[1] Briefly, the anode and cathode are commercial electrodes from DiOxide Materials, which utilised $NiFeO_x$ on SS316L fibre paper as OER electrode and Raney nickel nickel fibre paper as HER electrode. Both electrodes were created through the catalyst coated substrate method by hand painting on a catalytic ink using Nafion perfluorinated resin solution (5 wt.%) as binder as originally noted by their originators.[2] Similarly, the Sustainion X37-50 RT membranes were activated by 24 hours of submersion in a 1.0 M KOH solution. This solution was created by combining an appropriate mixture of potassium hydroxide reagent grade, 90%, flakes (Sigma Aldrich) and deionised water. The cell components were assembled in a top-down approach, where the

membrane was placed on top of the anode, followed by placing the cathode on top of the membrane again to form the complete MEA. This was repeated successively for each MEA. Further, a compressive force of 2 kN per spring was ensured based on the spring design and compression.

Similarly to our previous work,[1] the higher heating value (HHV) is calculated through Eq. S1.

$$\text{HHV efficiency} = \frac{E_{HHV}}{E_{cell}} \tag{S1}$$

The electrical efficiency at 1 kW power was determined as shown in Eq. S2, and avoids interpretative issues with HHV vs lower heating value.

$$\begin{aligned}\text{Electrical efficiency} &= \frac{zF}{IM_w} \cdot P \cdot \frac{1}{3.6\ MJ\ kWh^{-1}} \\ &= \frac{2 \cdot 96485\ C\ mol^{-1}}{500\ C\ s^{-1} \cdot 2.016 \times 10^{-3}\ kg_{H_2}\ mol^{-1}} \cdot 1000\ W \cdot \frac{1}{3.6\ MJ\ kWh^{-1}} \\ &= 53.18\ kWh\ kg_{H_2}^{-1}\end{aligned} \tag{S2}$$

1.2 System operation methodology

The 1 kW$_{el}$ AEMWE balance-of-plant (BoP) setup was a scaled-up version of the system used in single-cell AEM testing, as shown in Fig. S1a. The electrolyser was operated in a twin-electrolyte feed mode, with aqueous 1.0 M KOH circulated on both the hydrogen evolution reaction (HER) and oxygen evolution reaction (OER) sides. Electrolyte flow rates from the storage tanks were controlled using gear pumps and monitored with two mass flow meters. A flow rate of 1 mL min^{-1} cm^{-2} was maintained to prevent membrane dehydration.

The conductivity of the circulating electrolyte was continuously monitored to detect any changes in concentration. To minimise CO_2 contamination and evacuate dissolved gases, argon was periodically purged into the storage tanks. Refill pumps were employed to main-

tain a constant level of deionised (DI) water, visually monitored using level indicators. The electrolyte storage tanks were continuously mixed to stabilise pH during operation, as shown in Fig. S1b.

Thermal control of the stack was achieved using four 250W cartridge heaters embedded in the end plates. These were regulated via a PID control system and monitored using a type K thermocouple to maintain the desired operating temperature. Individual cell voltages were recorded, and the system was controlled using a PLC-based control unit. To prevent oxidation of the SS304 tubing on the OER side, the storage tank was protected using an impressed current cathodic protection (ICCP) system, as shown in Fig. S1c.

ICCP is a corrosion mitigation technique in which an external DC power source drives current through a shared electrolyte between a stable anode and the metal structure to be protected— in this case, the SS304 KOH storage tank.[3,4] ICCP forms an electrochemical cell that shifts the protected component's potential into the cathodic range, thereby preventing oxidation. The applied current must be carefully controlled, as excessive polarisation can lead to parasitic reactions such as hydrogen evolution due to water splitting. Cathodic potentials should therefore remain below critical thresholds to avoid unintended electrochemical side effects.

ICCP systems can be designed to offer partial protection—targeting specific components of the BoP —or complete protection, depending on the system configuration and electrolyte conductivity.[5,6] While partial protection may be sufficient in many cases, full protection ensures uniform corrosion mitigation but may require greater energy input and stricter control. The technique is widely adopted across various sectors including marine structures, offshore platforms, underground pipelines, water treatment systems, and reinforced concrete infrastructure.[4,6,7]

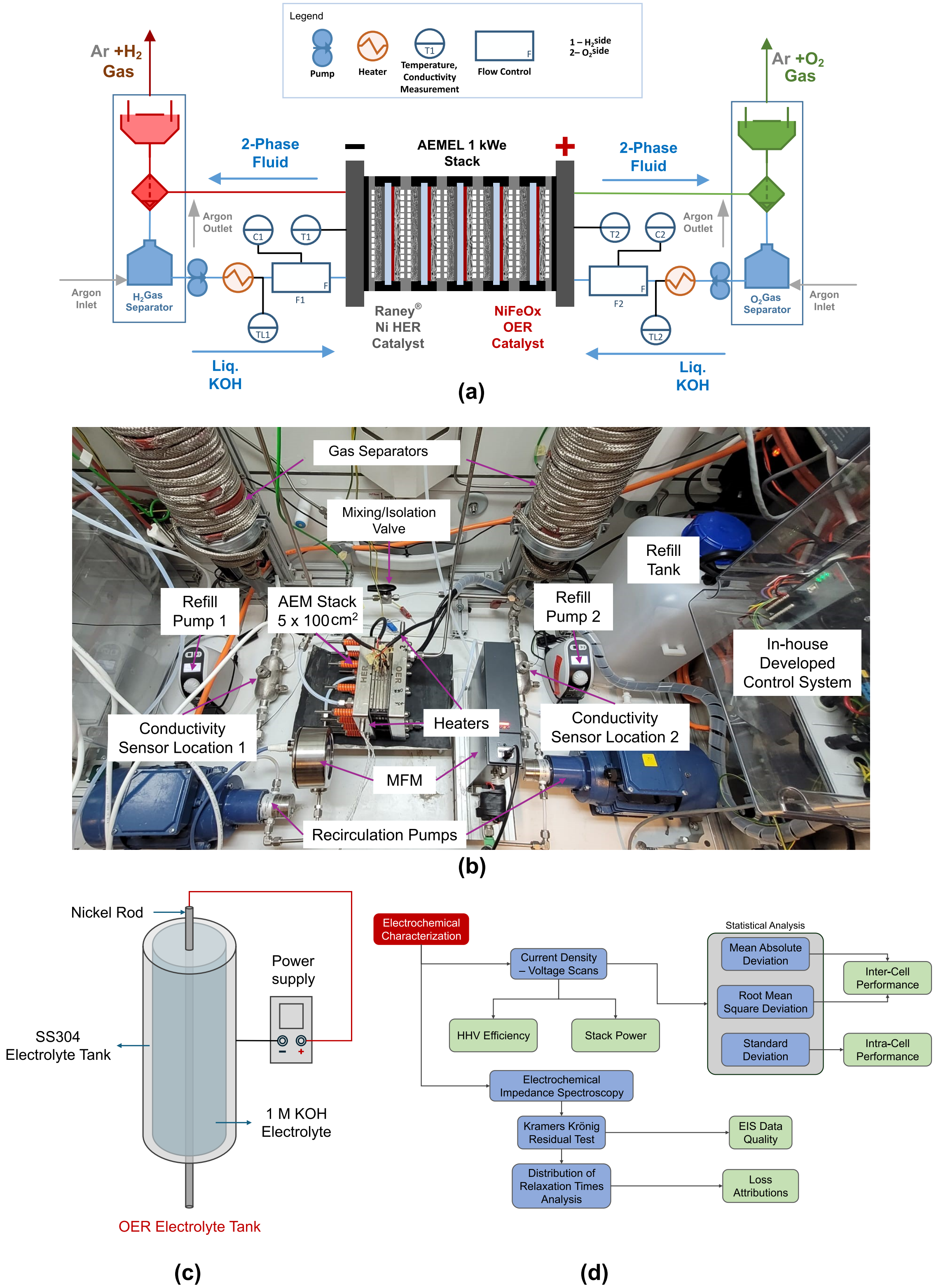

Figure S1: System design and analysis overview: **(a)** Schematic representation of the 5-cell, 100 cm^2 repeating unit AEMWE stack and associated balance of plant; **(b)** in-house developed assembly along with the patent-filed stack; **(c)** schematic of the impressed current cathodic protection system used to mitigate corrosion in the electrolyte tank; and **(d)** electrochemical and statistical analysis methodology employed for performance evaluation.

Its application in electrolysis systems demonstrates its versatility and relevance, particularly in environments where alkaline electrolytes accelerate corrosion. Proper ICCP implementation, combined with real-time monitoring, is therefore essential to ensure long-term durability without incurring the risks associated with overprotection.[3]

1.3 Electrochemical and statistical analysis methodology

The stack performance was characterised in parallel using current–voltage (JV) and electrochemical impedance spectroscopy (EIS) methods, employing a Zahner EL1000 loadbox, Zennium X potentiostat and a third-party power-supply unit. Twisted cables were used to minimise inductive effects on the measured spectra. Statistical analysis of the JV curve data was carried out to evaluate the stack's electrochemical performance and its response to varying current densities.

Further, EIS measurements were conducted at discrete current densities of 0.05, 0.1, and from 0.2 up to 1.0 A cm^{-2}, in increments of 0.1 A cm^{-2}, using a galvanostatic perturbation amplitude corresponding to 1–10% of the applied bias, as shown in Tab.S1. Data quality was verified using Kramers–Krönig relations, with a tolerance limit of ±2%.

Table S1: The EIS Amplitude summary as a percentage of Bias current across the galvanostatic measurements.

**Bias (A cm^{-2})**	**0.05**	**0.1**	**0.2**	**0.3**	**0.4**	**0.5**	**0.6**	**0.7**	**0.8**	**0.9**	**1**
**Bias (A)**	5	10	20	30	40	50	60	70	80	90	100
**Amplitude (A)**	0.5	1	1	1	1	1	1	4	4	4	10
**% of Bias**	**10.0**	**10.0**	**5.0**	**3.3**	**2.5**	**2.0**	**1.7**	**5.7**	**5.0**	**4.4**	**10.0**

Subsequently, distribution of relaxation times (DRT) analysis was performed using the Tikhonov regularisation method to decouple membrane–electrode assembly (MEA) losses. The overall methodology is illustrated in Fig. S1d.

Cell voltage was selected as the basis for statistical analysis, as it constituted the primary output from the electrochemical tests. To assess inter-cell performance, each cell's voltage was compared against the five-cell average using two metrics: the mean absolute deviation

(MAD) and the root mean square deviation (RMSD). MAD offers a robust measure of uniformity, as it quantifies the average absolute deviation from the mean without being disproportionately influenced by outliers. In contrast, RMSD assigns greater weight to larger deviations,making it more sensitive to cells exhibiting significant voltage divergence.

A third metric—the standard deviation (SD)—was employed to evaluate intra-cell performance. Unlike RMSD, which reflects deviation from the global average, SD captures the variability within each cell by measuring the spread of residuals relative to that cell's own mean voltage. Although it uses the same squared residuals as RMSD, the centring around the individual mean allows SD to characterise the internal consistency of each cell across the tested current densities.

The MAD was first calculated for each cell to assess the overall consistency of its voltage relative to the average voltage curve across all current densities, as shown in Eq. S3 .

$$MAD_i = \frac{1}{m}\sum_{j=1}^{m} |V_i(J_j) - \overline{V}(J_j)| \tag{S3}$$

Here, $V_i(J_j)$ denotes the voltage of cell i at current density J_j, and $\overline{V}(J_j)$ is the average voltage across all five cells at the same current density. The resulting MAD values (expressed in millivolts) provide a robust, unit-consistent measure of overall voltage consistency, offering insight into how uniformly each cell tracks the mean performance curve across all operating points.

To complement this, the root mean square deviation (RMSD) was computed for each cell to capture the magnitude of deviation from the stack average, as defined in Eq. S4.

$$RMSD_i = \sqrt{\frac{1}{m}\sum_{j=1}^{m}[V_i(J_j) - \overline{V}(J_j)]^2} \tag{S4}$$

RMSD, also expressed in millivolts, gives more weight to larger deviations, making it more sensitive to cells exhibiting occasional but significant divergence from the average. While

both MAD and RMSD assess inter-cell behaviour, RMSD tends to emphasise the worst-performing instances, whereas MAD provides a more balanced view.

To evaluate intra-cell consistency—how each individual cell behaves across the full range of current densities—the standard deviation (SD) of the residuals was calculated using Eq. S5.

$$SD_i = \sqrt{\frac{1}{m-1}\sum_{j=1}^{m}[r_{i,j} - \overline{r}_i]^2} \tag{S5}$$

In this expression, $r_{i,j} = V_i(J_j)$ - $\overline{V}(J_j)$ represents the voltage residual of cell i at current density J_j, and r_i is the mean residual for that cell. Unlike RMSD, which centres each residual around the stack-wide mean, SD focuses on the variation of each cell's residuals around its own mean. As such, it isolates the internal voltage fluctuation of a single cell and serves as a metric for intra-cell voltage stability, with lower SD values indicating more consistent cell performance across the range of current densities.

Based on the statistical analysis, a cell exhibiting moderate divergence from the average behaviour was selected for further EIS investigation, as it captures characteristics common to both the most irregular and the most consistent cells.

2. Results

2.1 ICCP and Temperature Ramp

The open-circuit potential (OCP) measured between the nickel anode and the SS304 tanks of the ICCP system was found to be +0.12 V. Upon immersion in 1.0 M KOH, the nickel rod develops a thin surface layer of nickel hydroxide ($Ni(OH)_2$), establishing a $Ni(OH)_2$/Ni redox couple. This electrochemical equilibrium is described by the reaction in Eq. S6.

$$Ni(OH)_2(s) + 2e^- \leftrightarrow Ni(s) + 2OH^- \tag{S6}$$

The standard potential for this reaction under alkaline conditions (pH 14) is E^0 = -0.72 V vs SHE.[8,9] However, the typical OCP of a Ni rod in 1.0 M KOH is approximately -0.36V vs SCE, which corresponds to –0.12 V vs SHE.[10]

An OCP of +0.12 V was recorded between the nickel rod and the SS304 tank (Fig. S2a), which equates to 0.00 V vs SHE. This indicates that the SS304 tank was already within the passive region. The potential domains for protection of SS304 in 1.0 M KOH are summarised in Tab. S2.

To ensure full protection, a cathodic current of –100 mA was applied. This polarised the SS304 to a potential of -0.3 V vs Ni, equivalent to -0.42 V vs SHE. Given that the Cr_2O_3 passive film is stable in the range of -0.5V to +0.8V vs SHE in 1.0 M KOH,[11,12] it can be concluded that complete cathodic protection was achieved. Notably, maximum protection is expected near –0.5 V vs SHE, which corresponds to -0.62 V vs the nickel reference.

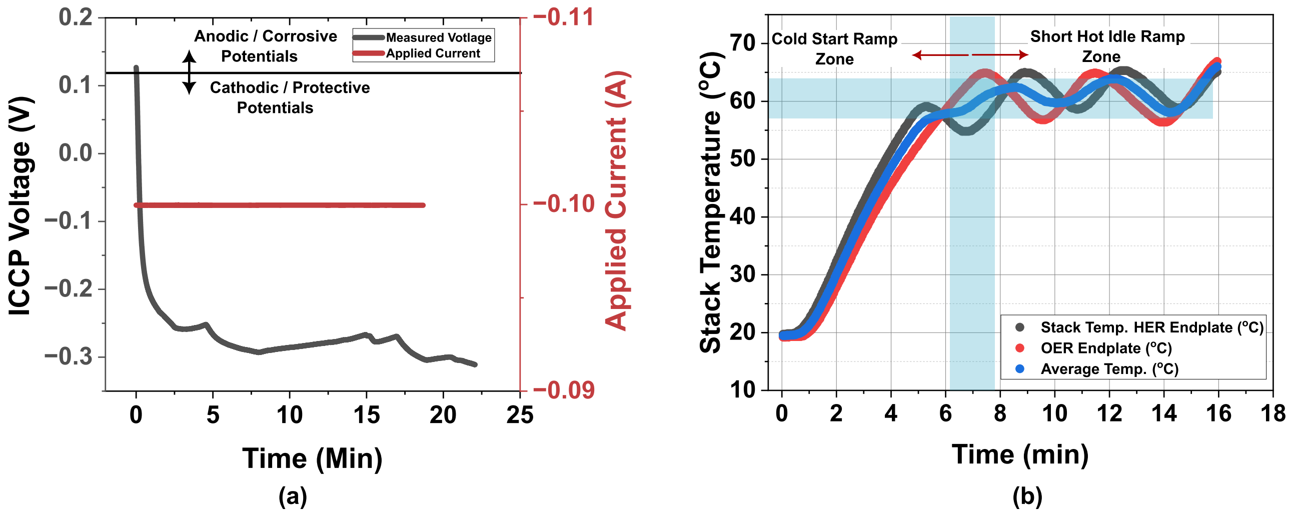

Figure S2: **(a)** Impressed current cathodic protection (ICCP) system employed to mitigate corrosion on the O_2 side of the 1.0 M KOH tank. **(b)** Cold-start and hot-idle start time intervals of the 1 kW_e AEMWE stack.

Following the assembly of the 5-cell AEMWE short stack, the cold-start and hot idle ramp durations were evaluated to assess the system's thermal response, as illustrated in Fig. S2b. Starting from ambient temperature, the system reached its operating temperature of 60°C after seven minutes.

Table S2: The protective and corrosion zones for SS304 in 1.0 M KOH solution.

**Region (vs SHE)**	**Behaviour**	**Corrosion?**
<–0.5 V	HER zone (cathodic protection)	None
–0.5 to +0.8 V	Cr_2O_3 film stable (passive)	None
>+0.8 V	Cr dissolves (transpassive)	Possible

During the cold-start ramp, the primary limitation was the thermal mass of the stack assembly, which governs the rate of temperature increase. In contrast, the hot idle ramp was limited by the rate of applied current, which influenced internal heat generation. Although the electrochemical reactions are exothermic at operating temperature, the system's substantial thermal mass, combined with the convective cooling effect of the circulating liquid electrolyte, was insufficient to independently raise the temperature to 60°C. As a result, external heating via cartridge heaters was necessary.

The heating profile was controlled using a PID controller. A time lag of approximately one minute was observed between the change in set-point and the actual temperature response. This delay is attributed to the relatively low thermal conductivity of stainless steel, which was used for the stack end plates. For reference, stainless steel (e.g., SS304) has a thermal conductivity of approximately 14–16 W $m^{-1}\cdot K^{-1}$,[13] whereas aluminium (e.g., 6061 alloy) offers a significantly higher value of 167–180 W $m^{-1}\cdot K^{-1}$.[14] This represents a thermal conductivity difference of over an order of magnitude, suggesting that the use of aluminium end plates could substantially reduce thermal lag and improve system responsiveness.

2.2 Statistical Analysis of Stack Performance

The polarisation curves for all five cells exhibit excellent overlap, reflecting a high degree of uniformity in electrochemical behaviour across the stack, as illustrated in Fig. 1a. Following the statistical framework outlined above, the deviation of each cell's voltage from the average jV-curve was statistically quantified to evaluate the inter-cell and intra-cell consistency. This was achieved through an analysis of squared residuals across the full range of current

densities.

Cell-level mean absolute deviation (MAD) analysis revealed distinct patterns of voltage behaviour. Cell 4 exhibited the lowest MAD (approximately 0.21 mV), confirming its highly consistent performance relative to the mean IV curve. Cell 1 followed closely with a MAD of 0.28 mV, while Cell 3, despite a moderately high root mean square deviation (RMSD), showed a comparatively low MAD of 0.74 mV, indicating consistently distributed deviations. Cell 5 displayed greater fluctuation, with a MAD of 1.39 mV, whereas Cell 2 showed the highest MAD at 3.66 mV, indicating the most erratic behaviour. These results highlight the utility of MAD in distinguishing between cells with uniformly small variations and those with larger or inconsistent voltage deviations.

The results also indicated that Cell 4 exhibited the smallest RMSD (approximately 14.2 mV), identifying it as the most consistent performer relative to the mean response. Cell 1 and Cell 3 followed with RMSD values of approximately 29.2 mV and 31.2 mV, respectively, indicating a moderate level of conformity. Cell 5, with an RMSD of 42.5 mV, displayed greater deviation, while Cell 2 exhibited the largest divergence from the mean, with an RMSD of 49.3 mV.

The standard deviation (SD) results revealed that Cell 4 exhibited the lowest SD (approximately 2.91 mV), indicating that it not only aligned closely with the 5-cell average voltage but also showed minimal internal fluctuation. Cell 1 followed with a relatively low SD of 0.39 mV, suggesting a stable performance with moderate internal consistency. Cell 3, despite its moderate RMSD, showed a higher SD (approximately 1.53 mV), indicating more internal variation, though still relatively stable compared to other cells. Cell 5 had a significantly higher SD of 10.51 mV, reflecting considerable internal fluctuations and less stable performance. Cell 2, with an SD of 0.37 mV, demonstrated the most erratic performance, with substantial variation within the cell's voltage behaviour. Although Cell 2 was intra-cell consistent, the variation in MAD and RMSD shows deviations higher than Cell 5, making Cell 2 more erratic in performance. These results underscore the importance of

SD in assessing the uniformity of each cell's response, highlighting internal deviations not captured by RMSD alone.

Together, the combined use of MAD, RMSD and SD enables a more nuanced understanding of inter-cell and intra-cell performance variation. The cell voltage deviations are represented in Tab. S3. Based on the statistical analysis and the corresponding ranking table, Cell 3 was chosen for AC–DC electrochemical analysis, as it exhibited intermediate behaviour and best represented individual cell performance within the stack.

Table S3: Ranking of individual cells based on MAD, RMSD, and SD from the 5-cell average voltage. Lower values indicate closer alignment with the average electrochemical behaviour and higher consistency.

Rank	Cell	MAD (mV)	RMSD (mV)	SD (mV)	Interpretation (with respect to the 5-cell average voltage)
1	Cell 4	0.21	14.2	2.91	Shows excellent alignment with the 5-cell average voltage, with minimal overall and internal deviation. SD indicates stable internal consistency.
2	Cell 1	0.28	29.2	0.39	High consistency with moderate deviation from the 5-cell average voltage and relatively low internal fluctuation. SD suggests moderate internal stability.
3	Cell 3	0.74	31.2	1.53	Moderate alignment with the 5-cell average voltage, but higher internal fluctuation indicates less stability. SD reflects these internal fluctuations.
4	Cell 5	1.39	42.5	10.51	Greater deviation from the 5-cell average voltage with significant internal fluctuation. SD shows high instability within the cell.
5	Cell 2	3.66	49.3	0.37	Largest deviation from the 5-cell average voltage, with significant and erratic voltage fluctuations. SD highlights considerable internal variation.

2.3 Electrochemical

Table S4: The DRT peaks association with processes based on our previous work.[1]

MEA Peaks	H_2 Half-cell Peaks	O_2 Half-cell Peaks	Frequency Range (Hz)	Process Description
P1	-	-	1- 5	Water and gas diffusion related process of HER, OER electrodes
P2	-	-	5 - 20	
P3	-	-	25 - 80	
P4	P4(i)H_2	P4(i)O_2, P4(ii)O_2	60 - 2000	HER, OER charge transfer
P5		P5O_2	2200-3200	OER ion transport
P5	P5H_2		7000-9000	HER ion transport

References

(1) Sampathkumar, S.; Ferriday, T.; Daviran, S.; Moussaoui, M.; Aubin, P.; Lawand, K.; Mensi, M.; Schouwink, P.; A., T.; Subotić, V.; Thévenot, A.; Dionigi, F.; Strasser, P.; Van Herle, J. Combinatorial Use of Reference Electrodes and DRT for disentangling AEM Electrolyser Losses. *ACS Energy & Fuels* **2025**,

(2) Liu, Z.; Sajjad, S.; Gao, Y.; Yang, H.; Kaczur, J.; Masel, R. The effect of membrane on an alkaline water electrolyzer. *International Journal of Hydrogen Energy* **2017**, *42*, 29661–29665.

(3) Christodoulou, C.; Glass, G.; Webb, J.; Austin, S.; Goodier, C. Assessing the long term benefits of Impressed Current Cathodic Protection. *Corrosion Science* **2010**, *52*, 2671–2679.

(4) Hussein Farh, H.; Ben Seghier, M.; Taiwo, R.; Zayed, T. Analysis and ranking of corrosion causes for water pipelines: A critical review. *NPJ Clean Water* **2023**, *6*, 65.

(5) Hamsir, H.; Sutresman, O.; Arsyad, H.; Syahid, M.; Widyianto, A. Suppression of corrosion on stainless steel 303 with automatic impressed current cathodic protection (a-ICCP) method in simulated seawater. *Eastern-European Journal of Enterprise Technologies* **2022**, *6*, 120.

(6) Dargahi, M.; Mahidashti, Z.; Rezaei, M. Corrosion prevention of storage tank bottom using impressed current cathodic protection–experimental and simulation study. *Engineering Failure Analysis* **2024**, *158*, 107982.

(7) Khudhair, A.; Hussein, F. Implementation of a control and monitoring system for a cathodic protection cell to mitigate localized corrosion in fixed and mobile steel structures. *Results in Engineering* **2024**, *23*, 102553.

(8) Bard, A.; Faulkner, L.; White, H. *Electrochemical Methods: Fundamentals and Applications*; John Wiley & Sons, 2022.

(9) Pourbaix, M. Atlas of electrochemical equilibria. *Aqueous Solution* **1966**,

(10) Franceschini, E.; Llorente, V.; Lanterna, A. Ni composite electrodes for hydrogen generation: Activation of Nb-based semiconductors. *international journal of hydrogen energy* **2022**, *47*, 15992–16004.

(11) Song, G. Transpassivation of Fe-Cr-Ni stainless steels. *Corrosion Science* **2005**, *47*, 1953–1987.

(12) Burleigh, T.; Dotson, T.; Dotson, K.; Gabay, S.; Sloan, T.; Ferrell, S. Anodizing steel in KOH and NaOH solutions. *Journal of The Electrochemical Society* **2007**, *154*, C579.

(13) Mirzababaei, S.; Doddapaneni, V.; Lee, K.; Paul, G.; Pirgazi, H.; Tan, K.-S.; Ertorer, O.; Chang, C.-H.; Paul, B.; Pasebani, S. Remarkable enhancement in thermal conductivity of stainless-steel leveraging metal composite via laser powder bed fusion: 316L-Cu composite. *Additive Manufacturing* **2023**, *70*, 103576.

(14) Nam, S.; Kim, S.; Kim, D.; Song, S.; Lee, J.; Kim, H.; Sung, H.; Choi, H. Enhancing thermal conductivity of 6061 Al plate via graphene dip coating. *Journal of Materials Research and Technology* **2024**, *29*, 3126–3134.
